# Risk factors for post-COVID-19 condition among previously hospitalized COVID-19 patients: a systematic review and meta-analysis

**DOI:** 10.3389/fpubh.2026.1911482

**Published:** 2026-08-14

**Authors:** Samal Kassymbek, Aigul Abduldayeva, Nikolay Safonov, Gulnur Doszhanova

**Affiliations:** Research Institute of Preventive Medicine named after Academician E. Dalenov, Astana Medical University, Astana, Kazakhstan

**Keywords:** hospitalization, long COVID, meta-analysis, post-COVID syndrome, post-COVID-19 condition, predictors, PRISMA, respiratory support

## Abstract

**Background:**

Post-COVID-19 condition is a major source of long-term morbidity after SARS-CoV-2 infection. Patients hospitalized for COVID-19 may be at particularly high risk because hospitalization often reflects greater acute disease severity and comorbidity burden. However, evidence on risk factors in previously hospitalized populations remains inconsistent due to differences in outcome definitions, follow-up periods, and analytical approaches. This systematic review and meta-analysis aimed to identify risk factors associated with post-COVID outcomes among patients previously hospitalized for COVID-19.

**Methods:**

A systematic review and meta-analysis was conducted following PRISMA 2020 principles. PubMed/MEDLINE, Scopus, Web of Science Core Collection, and the WHO COVID-19 Global Literature Database were searched for studies published between 1 January 2020 and 24 February 2026. Eligible studies included hospitalized patients with confirmed or probable COVID-19, assessed post-acute outcomes at least 4 weeks after acute infection, hospitalization, recovery, or discharge, and evaluated at least one candidate risk factor. Adjusted effect estimates were prioritized, and random-effects meta-analyses were performed when at least two comparable estimates were available.

**Results:**

Eleven studies were included in the qualitative synthesis, and seven contributed to quantitative meta-analysis. Five risk-factor groups were pooled: female sex, acute disease severity or respiratory support, intensive care unit (ICU) admission, vaccination status, and hypertension. Female sex was associated with higher odds of post-COVID outcomes (OR 1.94, 95% CI 1.60–2.35; *I*^2^ = 6.9%). The strongest association was observed for greater acute disease severity or advanced respiratory support (OR 2.55, 95% CI 1.77–3.68; *I*^2^ = 13.0%). ICU admission (OR 1.88, 95% CI 1.24–2.83), no or incomplete vaccination (OR 1.90, 95% CI 1.33–2.72), and hypertension (OR 1.60, 95% CI 1.16–2.20) were also associated with increased odds. Additional predictors, including obesity, age, smoking, dysgeusia, symptom burden, and neurological or psychiatric comorbidity, were summarized narratively.

**Conclusion:**

Among previously hospitalized COVID-19 patients, the most consistent risk factors for post-COVID outcomes were female sex, greater acute disease severity or respiratory support, ICU admission, no or incomplete vaccination, and hypertension. These findings support risk-based post-discharge follow-up, although the evidence remains limited by heterogeneous outcome definitions and the small number of studies available for several risk factors.

## Introduction

The coronavirus disease 2019 (COVID-19) pandemic has resulted in a large population of survivors who continue to experience health problems beyond the acute phase of SARS-CoV-2 infection. These post-acute manifestations may include persistent, relapsing, or newly developed respiratory, cardiovascular, neurological, cognitive, psychological, musculoskeletal, and functional symptoms. In the literature, these outcomes have been described using several overlapping terms, including Long COVID, post-COVID-19 condition (PCC), post-COVID syndrome, post-acute sequelae of SARS-CoV-2 infection (PASC), and persistent post-COVID symptoms (1–6). PCC can also be viewed within the broader landscape of post-acute infection syndromes, in which a subset of patients experience prolonged, fluctuating, or multisystem symptoms after an infectious trigger. This broader context is important because some clinical features of PCC overlap with post-viral fatigue states, dysautonomia-like syndromes, post-intensive-care sequelae, and delayed recovery after severe systemic illness. Nevertheless, the present review focuses specifically on post-COVID outcomes among patients hospitalized for COVID-19.

The terminology used to describe post-acute COVID-19 outcomes has evolved throughout the pandemic and remains incompletely standardized. The World Health Organization defines post-COVID-19 condition as a condition occurring in individuals with a history of probable or confirmed SARS-CoV-2 infection, usually 3 months from the onset of COVID-19, with symptoms lasting for at least 2 months and not explained by an alternative diagnosis (1, 2). Other clinical and public health frameworks use related but not identical terminology and time thresholds. For example, the Centers for Disease Control and Prevention describes Long COVID or Post-COVID Conditions as a broad range of ongoing, relapsing, or new health problems after SARS-CoV-2 infection, whereas the National Institute for Health and Care Excellence distinguishes ongoing symptomatic COVID-19 from 4 to 12 weeks and post-COVID-19 syndrome beyond 12 weeks (3–6).

This terminology problem is especially important for risk-factor research. In primary studies, the label used by the authors does not always correspond to a single operational diagnostic algorithm. Some studies define the outcome as WHO-PCC, others as at least one persistent symptom at a fixed follow-up time point, others as Long COVID, PASC, post-COVID syndrome, or patient-perceived non-recovery. These outcomes are closely related, but they are not methodologically identical. They may differ by time threshold, symptom duration, symptom list, requirement for functional impairment, attribution to SARS-CoV-2 infection, exclusion of alternative diagnoses, and method of symptom ascertainment. Accordingly, the terminology used by study authors and the operational case definition applied in each analysis were treated as separate variables. Author-reported terminology was not used alone to determine comparability across studies. Instead, we assessed whether studies used similar operational outcome definitions, follow-up thresholds, risk-factor constructs, and effect measures before considering quantitative pooling (7–10).

Patients hospitalized for COVID-19 represent a particularly important population for studying post-COVID risk. Hospitalization is a marker of more severe acute disease and is often associated with older age, comorbidity, higher inflammatory burden, respiratory failure, thromboinflammatory complications, immobility, intensive care exposure, and the need for oxygen therapy, non-invasive ventilation, or invasive mechanical ventilation. Persistent symptoms after discharge may therefore reflect several overlapping pathways: direct post-viral sequelae, residual pulmonary or cardiovascular injury, consequences of critical illness, deconditioning, neuropsychological stress, and worsening of pre-existing chronic conditions (11–14). This makes the hospitalized population clinically important, but also methodologically complex.

Early longitudinal cohorts of patients discharged after COVID-19 hospitalization showed that persistent symptoms and functional impairment could remain common for months after the acute episode. Studies from Wuhan demonstrated that fatigue or muscle weakness, sleep difficulties, anxiety or depression, reduced exercise capacity, and pulmonary diffusion impairment may persist at 6 and 12 months after discharge, with more severe acute illness associated with worse long-term outcomes in several domains (11, 12). Subsequent multicentre studies, including the PHOSP-COVID cohort in the United Kingdom, confirmed that many previously hospitalized patients did not perceive themselves as fully recovered months after discharge and that post-COVID morbidity may involve physical, cognitive, psychological, and functional components (13).

A growing number of studies have examined factors associated with the development of PCC, Long COVID, or persistent post-COVID symptoms after hospitalization. Female sex has repeatedly been associated with higher odds of persistent symptoms or incomplete recovery in post-discharge cohorts (13–17). Markers of acute COVID-19 severity, including intensive care unit admission, invasive mechanical ventilation, high-flow oxygen or non-invasive ventilation, severe-critical acute illness, and longer hospitalization, have also been reported as predictors of persistent symptoms or poorer recovery (13, 18–20). Several comorbidities, including obesity, hypertension, diabetes, chronic respiratory disease, cardiovascular disease, and neuropsychiatric conditions, have been evaluated as candidate risk factors, although their associations are less consistent and may be confounded by age, acute disease severity, baseline symptom burden, and healthcare-seeking behavior (13–20).

Vaccination status is another potentially important determinant of post-COVID outcomes. Evidence from broader infected populations suggests that vaccination before SARS-CoV-2 infection may reduce the risk of Long COVID, and some hospitalized cohorts have also reported lower risk of persistent symptoms among fully vaccinated patients compared with unvaccinated or incompletely vaccinated patients (16, 19, 21, 22). However, hospital-based evidence remains more limited than evidence from general infected populations. In addition, differences in pandemic period, circulating SARS-CoV-2 variants, admission criteria, treatment standards, and vaccine coverage may influence both the occurrence of post-COVID symptoms and the probability that they are detected or reported.

Although previous systematic reviews have synthesized the prevalence, symptom spectrum, and general burden of Long COVID, fewer reviews have focused specifically on risk factors among patients hospitalized for COVID-19. This distinction is important because risk factors in hospitalized populations may differ from those in community-based or predominantly mild infections. In hospitalized cohorts, predictors such as intensive care admission, respiratory support, and acute disease severity are clinically meaningful for post-discharge planning, whereas some population-level predictors may not have the same interpretation after conditioning on hospitalization. Furthermore, risk-factor studies differ substantially in outcome definitions, follow-up duration, adjustment variables, and reported effect measures, which complicates direct comparison and quantitative synthesis.

Identifying risk factors for post-COVID-19 condition among previously hospitalized patients has direct clinical and public health relevance. At the clinical level, risk stratification can help identify patients who may benefit from structured follow-up, rehabilitation, respiratory and cardiovascular evaluation, cognitive and mental health screening, and multidisciplinary care. At the health-system level, evidence on risk factors can support the development of post-discharge pathways, prioritization of high-risk groups, and planning of services for patients recovering from severe COVID-19.

The primary objective of this systematic review and meta-analysis was to identify and synthesize risk factors associated with the development of post-COVID-19 condition, Long COVID, PASC, post-COVID syndrome, persistent post-COVID symptoms, or incomplete recovery among patients hospitalized for COVID-19. Secondary objectives were to describe how post-COVID outcomes were defined across included studies, to distinguish author-reported terminology from operational outcome definitions, and to quantitatively synthesize key risk factors when sufficiently comparable adjusted effect estimates were available. This review aimed to provide an evidence base for risk-based post-discharge follow-up and organizational approaches to care for patients recovering after COVID-19 hospitalization.

## Methods

### Study design and reporting framework

This systematic review and meta-analysis was conducted in accordance with the Preferred Reporting Items for Systematic Reviews and Meta-Analyses 2020 statement (23). The reporting of the literature search was structured according to PRISMA-S recommendations, including transparent reporting of information sources, search dates, search terms, and database-specific search strategies (24).

The aim of this review was to identify and synthesize risk factors associated with the development of post-COVID-19 condition, Long COVID, post-COVID syndrome, post-acute sequelae of SARS-CoV-2 infection, or persistent post-COVID symptoms among patients previously hospitalized for COVID-19.

### Research question and eligibility framework

The review question was structured using an adapted Population-Exposure-Outcome framework. The population of interest included patients of any age and sex who had been hospitalized for confirmed or probable COVID-19. The exposure was hospitalization for COVID-19 and the subsequent post-acute follow-up period. The outcome was post-COVID-19 condition, Long COVID, post-COVID syndrome, PASC, persistent post-COVID symptoms, or incomplete recovery after COVID-19 hospitalization, as defined by the original studies.

The review question was: which demographic, clinical, comorbid, acute-severity, intensive-care, respiratory-support, vaccination-related, or other patient-level factors are associated with the development of post-COVID-19 condition, Long COVID, persistent post-COVID symptoms, or incomplete recovery among patients hospitalized for COVID-19?

### Search strategy

A systematic literature search was conducted in PubMed/MEDLINE, Scopus, Web of Science Core Collection, and the WHO COVID-19 Global Literature Database. The search covered studies published from 1 January 2020 to 24 February 2026. The complete database-specific executable search strategies are provided in Supplementary Table S1. Citation searching was performed using the reference lists of all studies included after full-text assessment and relevant systematic reviews identified during screening. Additional records identified through citation searching were considered for full-text assessment if their title or abstract indicated hospitalized or post-discharge COVID-19 patients, post-acute COVID outcomes, and risk-factor or predictor analysis. The final search was conducted on 24 February 2026. Studies published in English or Russian were eligible for inclusion. No restrictions were applied by country, age, or sex at the search stage. The language restriction was considered a potential source of language bias and was taken into account when interpreting the findings.

The search strategy combined controlled vocabulary terms, where available, and free-text terms related to SARS-CoV-2 infection, post-COVID outcomes, hospitalization, and risk factors. Search terms included combinations of COVID-19, SARS-CoV-2, coronavirus disease 2019, post-COVID-19 condition, post COVID-19 condition, Long COVID, post-COVID syndrome, PASC, persistent symptoms, post-acute sequelae, hospitalized, hospitalized, hospitalization, hospitalization, discharged, ICU, intensive care, risk factor, predictor, determinant, association, odds ratio, and multivariable analysis.

Studies were eligible if they provided primary data on patients hospitalized for COVID-19 or a clearly separable hospitalized subgroup. The search strategy combined the main post-COVID outcome terms with hospitalization-related and risk-factor-related terms across all databases, with database-specific syntax adapted where necessary.

### Eligibility criteria

Studies were eligible for inclusion if they met all of the following criteria: included patients with confirmed or probable SARS-CoV-2 infection; included patients hospitalized for COVID-19 or reported a clearly separable hospitalized subgroup; assessed post-COVID-19 condition, Long COVID, post-COVID syndrome, PASC, persistent post-COVID symptoms, new or persistent symptoms, or incomplete recovery after the acute phase; evaluated at least one candidate risk factor or predictor of the post-COVID outcome; reported an effect estimate with a measure of uncertainty or sufficient information for qualitative interpretation; used an observational cohort, cross-sectional follow-up, registry-based, case-control, or ambidirectional cohort design; and reported original primary data.

Confirmed and probable COVID-19 cases were considered eligible because several early-pandemic hospital cohorts were conducted during periods of limited testing availability, changing diagnostic capacity, and high community transmission, when clinical-radiological diagnosis was commonly used in hospital practice. Where reported, laboratory-confirmation status was extracted. However, because confirmation status was not consistently reported across all included studies, a sensitivity analysis restricted to laboratory-confirmed cases was not feasible.

The minimum eligible follow-up threshold was at least 4 weeks after acute COVID-19 onset, hospital admission, recovery, or hospital discharge, depending on how follow-up was defined in the original study. This broad threshold was used because early studies of post-COVID outcomes frequently defined persistent symptoms from 4 weeks onward. However, the operational definition used in each study was extracted separately, because this threshold is broader than the WHO clinical case definition of post-COVID-19 condition, which usually requires symptoms occurring 3 months from the onset of COVID-19 and lasting for at least 2 months, without an alternative diagnosis (1, 2).

Studies were excluded if they were case reports, small case series without analytic risk-factor data, narrative reviews, editorials, commentaries, letters without original data, protocols, modeling studies without primary observational data, or duplicate reports from the same cohort without new relevant risk-factor information. Studies were also excluded if they assessed only a single organ-specific abnormality without reporting an overall post-COVID syndrome-level outcome, or if they included a general infected population without separable data for hospitalized patients.

### Study selection

All retrieved records were imported into reference management software, and duplicate records were removed before screening. Titles and abstracts were screened for relevance to the review question. Potentially eligible reports underwent full-text assessment against the inclusion and exclusion criteria. Two reviewers independently screened titles and abstracts and assessed potentially eligible full-text reports. Disagreements were resolved through discussion and consensus. When consensus could not be reached, a third reviewer was consulted.

Studies were included in the qualitative synthesis if they provided primary data on risk factors or predictors of post-COVID outcomes among patients hospitalized for COVID-19. Studies were included in the quantitative synthesis if they reported comparable effect estimates with 95% confidence intervals for a risk factor reported by at least two studies.

**Figure 1:**
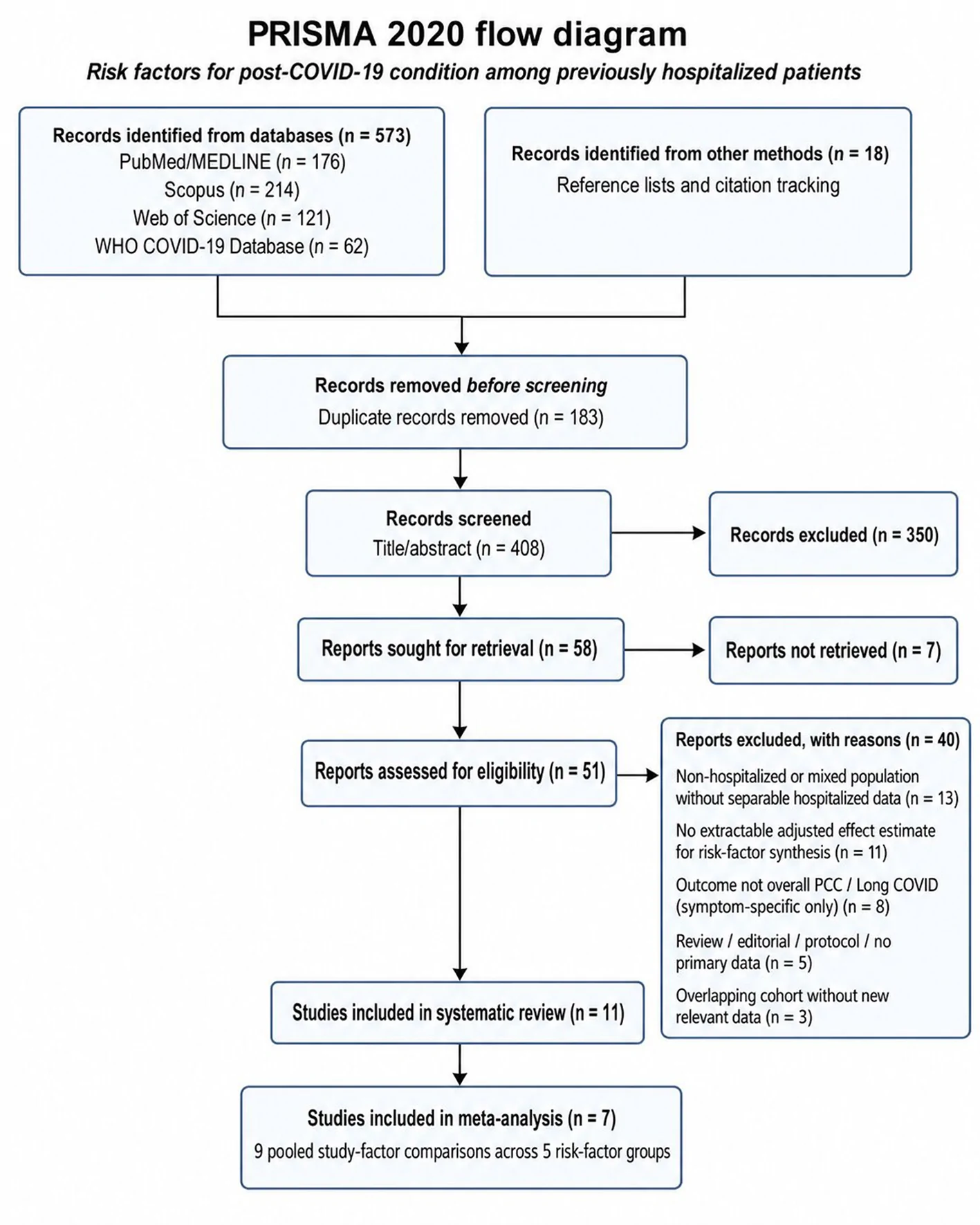
PRISMA 2020 flow diagram of study selection for the systematic review and meta-analysis of risk factors for post-COVID syndrome among previously hospitalized patients.

The study selection process is presented in the PRISMA 2020 flow diagram shown in Figure 1 (23).

### Data extraction

Data were extracted using a standardized extraction form developed for this review. Data extraction was performed independently by two reviewers. Extracted data were compared for consistency, and discrepancies were resolved by discussion and consensus before quantitative synthesis. Extracted study-level variables included author and year of publication, country, study design, population, analytic sample size, recruitment or data-collection period where reported, follow-up duration, post-COVID outcome label, operational outcome definition, confirmation status of acute COVID-19 where available, candidate risk factors, treatment-related or hospital-management variables where reported, and risk-of-bias category.

For each included study, the terminology used by the authors and the operational case definition applied in the analysis were extracted separately. Author labels included post-COVID-19 condition, Long COVID, post-COVID syndrome, PASC, persistent symptoms, new or persistent symptoms, and incomplete recovery. Operational definitions included WHO post-COVID-19 condition, at least one persistent symptom at a defined follow-up point, Long COVID defined by structured interview, persistent symptoms lasting at least 4 weeks, persistent symptoms assessed at three or more months, and patient-perceived incomplete recovery.

For risk-factor extraction, the following variables were collected where available: candidate risk factor, comparison group, effect measure, point estimate, lower and upper 95% confidence interval, follow-up time point, post-COVID outcome definition, adjustment status, and eligibility for pooled analysis. Adjusted estimates were prioritized over unadjusted estimates. When more than one adjusted estimate was reported for the same risk factor, the estimate corresponding to the main overall post-COVID outcome and the most complete adjustment model was selected.

### Risk-factor grouping and effect-estimate harmonization

Candidate risk factors were grouped according to clinical and methodological comparability before quantitative synthesis. The main prespecified risk-factor groups were female sex, acute disease severity or respiratory support, ICU admission, vaccination status, and comorbid conditions. A factor was considered eligible for meta-analysis when at least two studies reported sufficiently comparable effect estimates with 95% confidence intervals for the same risk-factor construct.

Effect directions were harmonized so that values above 1.0 consistently represented higher odds or risk of the adverse post-COVID outcome. When a study reported an odds ratio for full recovery, the estimate was inverted to represent incomplete recovery. When vaccination was reported as protective, the comparison was harmonized as no or incomplete vaccination vs. full vaccination where appropriate. For example, estimates for full vaccination as a protective factor were transformed so that the pooled comparison reflected higher risk among unvaccinated or incompletely vaccinated patients.

Adjusted odds ratios were the preferred effect measure for quantitative synthesis. Risk ratios, hazard ratios, and incidence rate ratios were extracted for qualitative synthesis but were not combined with odds ratios unless the effect measure and interpretation were sufficiently comparable. Unadjusted estimates were not combined with adjusted estimates in the primary pooled analyses. Treatment-related and hospital-management variables were extracted when reported, including corticosteroid use, antiviral or immunomodulatory treatment, anticoagulation, respiratory-support modality, ICU admission, and length of hospital stay. These variables were not pooled as a separate treatment-effect domain because they were inconsistently reported, clinically heterogeneous, and frequently confounded by indication and acute disease severity. Therefore, they were retained for qualitative interpretation when relevant.

### Handling of multiple estimates from the same study

Some studies reported multiple follow-up time points, multiple post-COVID outcomes, or several candidate risk factors. For qualitative synthesis, all relevant estimates were extracted and considered. For quantitative synthesis, only one estimate per study per risk-factor group was retained unless the estimates represented clearly distinct risk factors.

When several time points were available for the same risk factor, the estimate corresponding to the primary follow-up time point of the original study was prioritized. For studies using WHO post-COVID-19 condition, follow-up points at or beyond 3 months were prioritized. For broader persistent-symptom outcomes, the main post-acute follow-up time point defined by the study authors was retained. Repeated estimates from the same cohort were not combined within the same pooled risk-factor analysis to avoid double counting.

### Quality assessment

The methodological quality of included observational studies was assessed using a Newcastle-Ottawa Scale-based approach adapted for cohort and follow-up studies (25). The assessment included four domains: selection of the study population, comparability or representativeness, outcome assessment, and follow-up or response completeness.

Risk-of-bias assessment was performed independently by two reviewers. Disagreements in domain-level judgments or overall risk categories were resolved through consensus. The Newcastle-Ottawa Scale-based ratings were interpreted cautiously because observational post-COVID studies differed substantially in design, follow-up completeness, outcome ascertainment, and adjustment for confounding. Each study was assigned a total score and categorized as low-moderate, moderate, or moderate-high risk of bias. The main concerns considered during quality assessment included selection bias, loss to follow-up, self-reported outcome assessment, telephone or questionnaire-based symptom ascertainment, broad outcome definitions, limited adjustment for confounding, and difficulty distinguishing post-COVID symptoms from pre-existing conditions or background symptoms.

### Statistical analysis

Meta-analyses were performed separately for each risk factor when at least two studies reported sufficiently comparable effect estimates. The primary effect measure for quantitative synthesis was the adjusted odds ratio. Adjusted estimates were used because candidate risk factors such as age, sex, comorbidity, acute disease severity, respiratory support, ICU admission, and vaccination status are interrelated.

For each eligible odds ratio, the natural logarithm of the effect estimate was calculated. Standard errors were derived from the 95% confidence intervals using the formula:


$$\begin{array}{l} \text{SE }=\left(\text{ln upper CI }-\text{ ln lower CI}\right)\;/\;3.92. \end{array}$$

Pooled estimates were calculated using random-effects meta-analysis. The DerSimonian-Laird estimator was used to estimate between-study variance (26). Pooled effects were back-transformed from the log scale and reported as odds ratios with 95% confidence intervals.

The following risk-factor groups were eligible for quantitative synthesis: female sex, acute disease severity or respiratory support, ICU admission, vaccination status, and hypertension. Other candidate factors, including obesity, age, smoking, diabetes, dysgeusia, initial symptom load, neurologic or psychiatric comorbidity, and organ-specific abnormalities, were summarized narratively because the available effect estimates were not sufficiently complete or comparable for meta-analysis.

Statistical heterogeneity was assessed using Cochran's Q statistic, the *I*^2^ statistic, and tau-squared (27). *I*^2^ was interpreted as the proportion of total variability attributable to between-study heterogeneity rather than sampling error. Because several pooled analyses included only a small number of studies, heterogeneity estimates were interpreted cautiously.

Leave-one-out sensitivity analyses were performed for each pooled risk-factor group by sequentially excluding one study-effect estimate and recalculating the pooled odds ratio. These analyses were used to assess whether the direction or magnitude of association was driven by a single study.

Small-study effects and publication bias were planned to be assessed using funnel plot visualization and Egger's regression test when at least 10 studies were available for a given risk factor (28). Because fewer than 10 studies were available for each pooled risk-factor group, formal publication-bias testing was not performed. Funnel plots, if generated, were interpreted descriptively only.

Subgroup analyses were planned according to operational outcome definition, follow-up duration, age group, and geographic region if sufficient data were available. Because most risk-factor groups contained only a small number of studies, formal subgroup meta-analysis and meta-regression were not performed as primary analyses. Instead, differences in operational outcome definitions and follow-up duration were considered qualitatively when interpreting the pooled results.

All statistical analyses were conducted on the log odds ratio scale using validated meta-analysis procedures in R statistical software (29, 30). Statistical significance was assessed using two-sided tests, with *p* < 0.05 considered statistically significant where applicable. Interpretation emphasized effect size, direction of association, confidence intervals, consistency across studies, heterogeneity, and clinical plausibility rather than *p*-values alone.

## Results

### Study selection

The systematic search identified 573 records from electronic databases and 18 additional records through reference lists and citation tracking. After removal of 183 duplicate records, 408 records were screened by title and abstract. Of these, 350 records were excluded as not relevant to the review question. 58 reports were sought for retrieval, and seven could not be retrieved. 51 full-text reports were assessed for eligibility. Forty reports were excluded because they did not include a separable hospitalized population, did not provide extractable risk-factor estimates, did not report an overall post-COVID syndrome-level outcome, were not primary research reports, or represented overlapping cohorts without new relevant data. Finally, 11 studies were included in the qualitative synthesis, and 7 studies contributed effect estimates to the quantitative meta-analysis.

### Study characteristics

The 11 included studies were conducted in the United Kingdom, Russia, Brazil, Italy, Thailand, France, South Africa, and China. Most studies used prospective cohort or ambidirectional cohort designs, although cross-sectional follow-up and clinic-based follow-up designs were also represented. Analytic sample sizes ranged from 295 to 2,320 participants for the main follow-up samples, with one national cohort including 1,873 participants at 3 months after discharge. Follow-up duration ranged from approximately 54 days to 2 years after acute COVID-19 or hospital discharge. The characteristics of the included studies are summarized in Table 1.

**Table 1:** Characteristics of studies included in the systematic review.

References	Country	Sample/follow-up	Post-COVID definition	Main risk factors
PHOSP-COVID Collaborative Group, (13)	United Kingdom	2,320 at 5 months; 804 at 1 year	Patient-perceived incomplete recovery/multidomain impairment	Female sex, obesity, invasive mechanical ventilation, acute severity
Pazukhina et al. (15)	Russia	Adults *n* = 1,013; children *n* = 360; 6 and 12 months	WHO post-COVID-19 condition	Female sex, hypertension, comorbidities; pediatric neurologic and allergic respiratory conditions
Nascimento et al. (16)	Brazil	*n* = 412; 90 days	At least one persistent symptom	Female sex, ICU admission, incomplete or no vaccination
Rigoni et al. (18)	Italy	*n* = 413; 6 and 12 months	Any persistent symptom	High-flow oxygen, non-invasive ventilation, or invasive mechanical ventilation
Lersritwimanmaen et al. (19)	Thailand	*n* = 295; 3 months, 1 year, and 2 years	Long COVID by structured interview	Severe-critical acute illness, vaccination status
Bai et al. (17)	Italy	*n* = 377; more than 4 weeks after recovery	At least one physical or psychological symptom	Female sex, age, active smoking, acute severity markers
de Oliveira et al. (20)	Brazil	*n* = 439; 2–12 months after onset	At least one symptom lasting at least 4 weeks	ICU admission, dysgeusia
Ko et al. (33)	France	*n* = 316; 4 months after hospitalization	Persistent symptoms/Long COVID	Female sex, hypertension, number of initial symptoms
Dryden et al. (34)	South Africa	*n* = 1,873; 3 months after discharge	New or persistent symptoms	Female sex, ICU admission, oxygen therapy-related markers
Mandal et al. (14)	United Kingdom	*n* = 384; median 54 days after discharge	Persistent symptoms with biomarker and imaging follow-up	Persistent fatigue, dyspnea, inflammatory markers, abnormal imaging
Huang et al. (11)	China	*n* = 1,733; approximately 6 months after symptom onset	Persistent symptoms and domain-specific sequelae	Acute disease severity, female sex, age, organ-specific outcomes

Outcome definitions varied across studies. Some studies used WHO post-COVID-19 condition, whereas others defined the outcome as at least one persistent symptom, structured interview-defined Long COVID, new or persistent symptoms, or patient-perceived incomplete recovery. Because the review focused on risk factors, author-reported labels and operational case definitions were interpreted separately.

### Outcome terminology and operational definitions

Outcome terminology and operational definitions varied substantially across the included studies. Author-reported labels included post-COVID-19 condition, Long COVID, post-COVID syndrome, persistent symptoms, new or persistent symptoms, and incomplete recovery. These labels were related to the broad post-acute COVID-19 construct, but they did not correspond to a single uniform diagnostic algorithm across studies.

Operational definitions also differed. Some studies applied the WHO post-COVID-19 condition definition, whereas others defined the outcome as at least one persistent symptom at a specified follow-up time point, structured interview-defined Long COVID, persistent symptoms lasting at least 4 weeks, symptoms assessed three or more months after acute infection or discharge, or patient-perceived incomplete recovery. These definitions differed by time threshold, symptom duration, requirement for functional impairment, attribution to SARS-CoV-2 infection, exclusion of alternative diagnoses, and method of symptom ascertainment.

Because the objective of this review was to synthesize risk factors rather than prevalence alone, this heterogeneity was especially important. Risk-factor associations may differ depending on whether the outcome is defined as any persistent symptom, WHO-defined post-COVID-19 condition, multidomain impairment, or incomplete recovery. Therefore, author-reported terminology and operational outcome definitions were extracted separately. Pooled analyses were performed only when the risk-factor construct, effect measure, and outcome definition were considered sufficiently comparable for quantitative synthesis. Where comparability was limited, findings were retained for narrative interpretation rather than pooled meta-analysis.

### Risk-of-bias assessment

Risk-of-bias assessment indicated that most included studies had low-moderate or moderate risk of bias. The main methodological concerns were loss to follow-up, self-reported or telephone-based outcome assessment, broad symptom definitions, incomplete adjustment for confounding, and difficulty distinguishing post-COVID symptoms from pre-existing conditions or background symptoms. Three studies were rated as low-moderate risk, five as moderate risk, and three as moderate-high risk. Three studies were rated as low-moderate risk, five as moderate risk, and three as moderate-high risk (Table 2).

**Table 2:** Summary of Newcastle-Ottawa scale-based risk-of-bias assessment.

Risk category	Number of studies	Studies	Main concerns
Low-moderate	3	PHOSP-COVID (13) Dryden (34); Huang (11)	Attrition and/or self-reported outcomes but generally strong cohort design
Moderate	5	Pazukhina (15); Nascimento (16); Rigoni (18); Lersritwimanmaen (19); Ko (33)	Moderate follow-up limitations, self-report, and residual confounding
Moderate-high	3	Bai (17); de Oliveira (20); Mandal (14)	Broader outcome definitions, clinic/cross-sectional follow-up, or limited comparability

### Quantitative synthesis of risk factors

**Figure 2:**
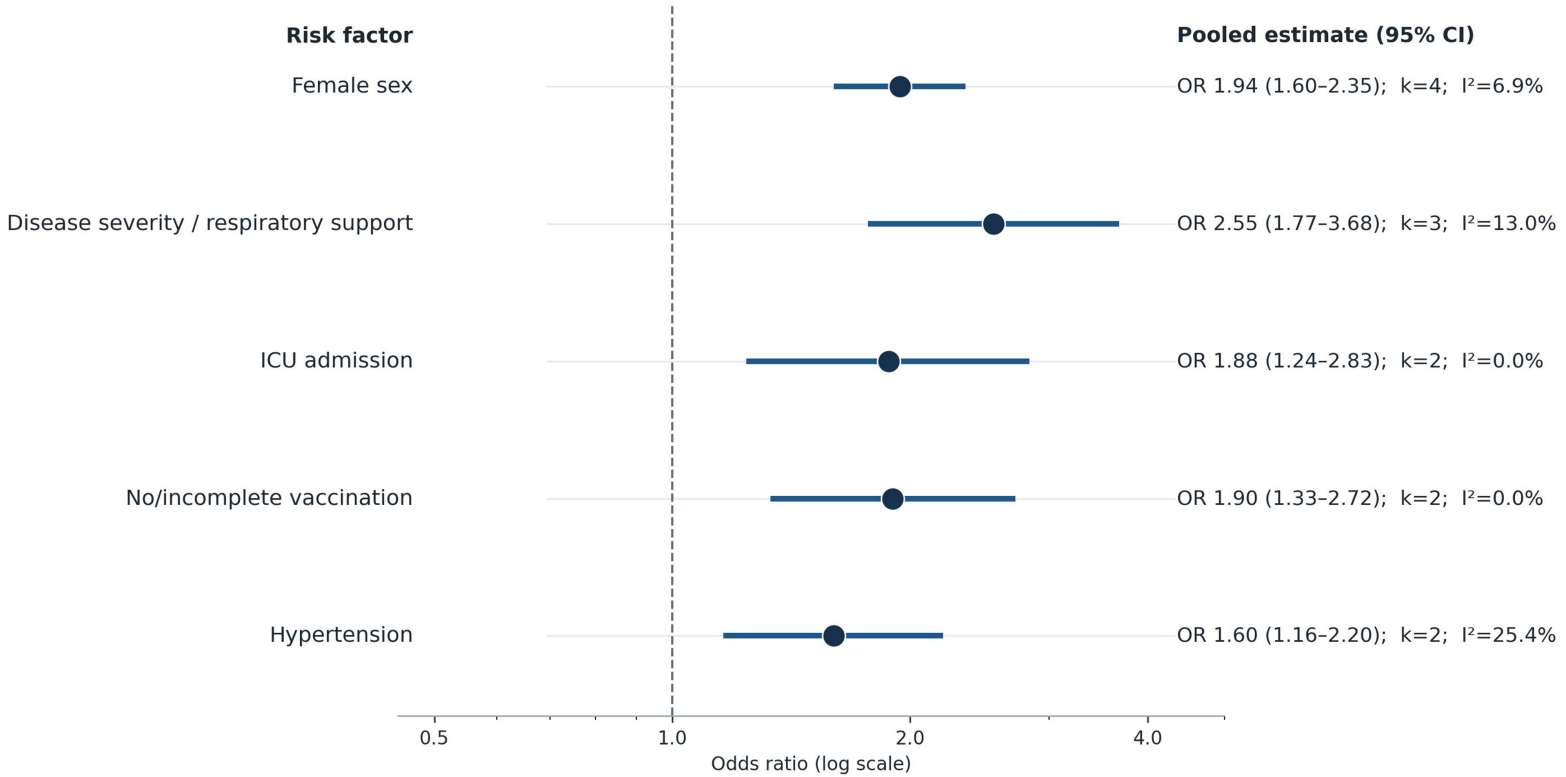
Summary forest plot of pooled associations between candidate risk factors and post-COVID syndrome among previously hospitalized patients.

**Figure 3:**
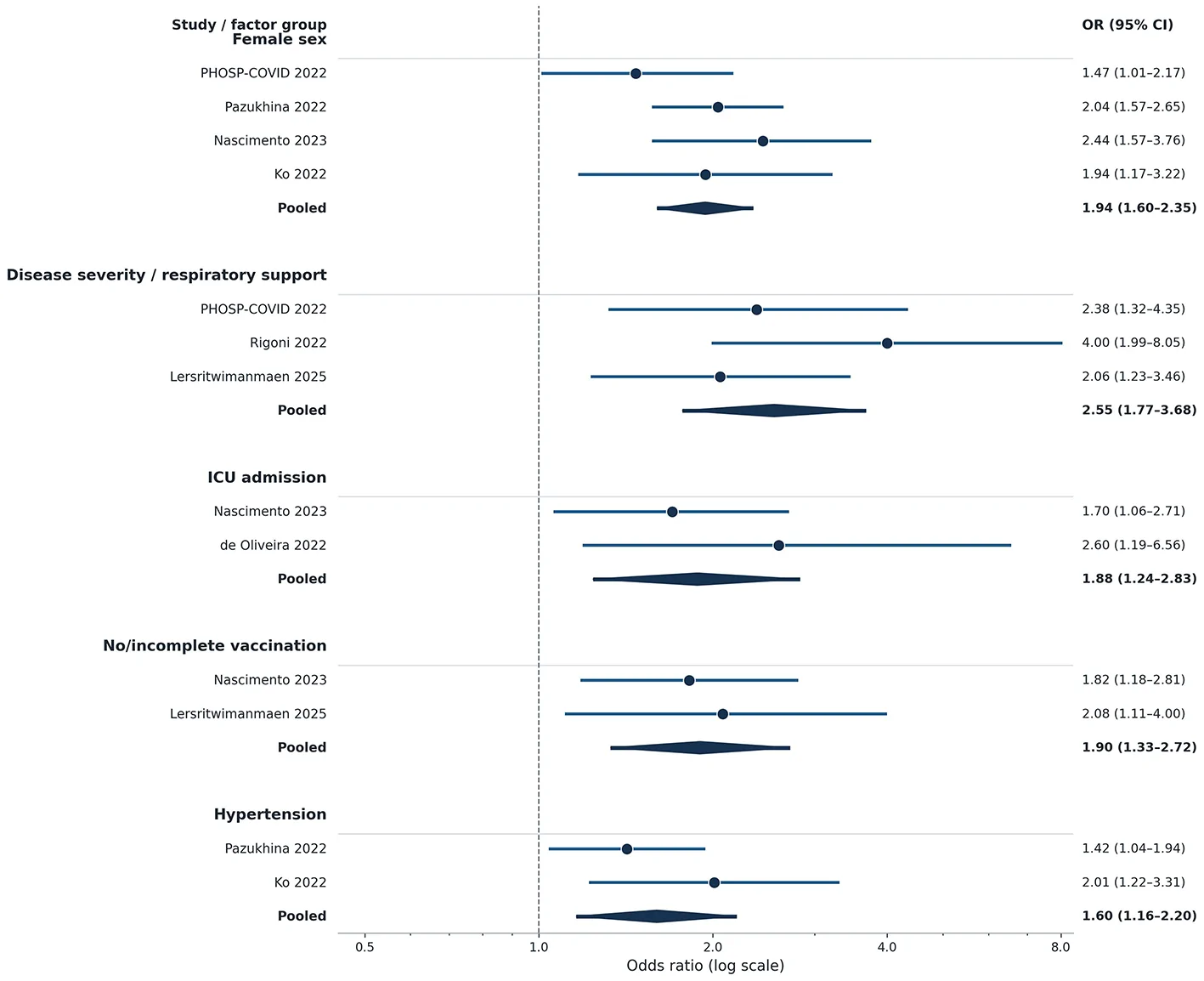
Study-level and pooled odds ratios for the five risk-factor groups included in the quantitative synthesis.

Five risk-factor groups were eligible for quantitative synthesis: female sex, acute disease severity or respiratory support, ICU admission, vaccination status, and hypertension. The pooled associations for the five risk-factor groups are summarized in Figure 2. Across all pooled analyses, effect estimates were harmonized so that odds ratios above 1.0 represented higher odds of the adverse post-COVID outcome. Protective effects, including vaccination, were inverted when necessary to provide a consistent risk-oriented interpretation. Study-level and pooled estimates for all five risk-factor groups are presented in Figure 3. A summary of the pooled estimates is provided in Table 3.

**Table 3:** Summary of pooled random-effects meta-analyses by risk-factor group.

Risk factor	*K*	Pooled OR	95% CI	I^2^, %	Tau^2^	Interpretation
Female sex	4	1.94	1.60–2.35	6.9	0.0028	Higher odds of post-COVID outcome among women
Disease severity/respiratory support	3	2.55	1.77–3.68	13.0	0.0139	Stronger association; clinically consistent with severe acute COVID-19
ICU admission	2	1.88	1.24–2.83	0.0	0.0000	Higher odds among patients requiring ICU care
No/incomplete vaccination	2	1.90	1.33–2.72	0.0	0.0000	Higher odds compared with fully vaccinated patients
Hypertension	2	1.60	1.16–2.20	25.4	0.0153	Association present, but evidence base limited

### Female sex

Four studies contributed to the pooled analysis of female sex. Female sex was associated with higher odds of post-COVID syndrome or incomplete recovery after COVID-19 hospitalization (pooled OR 1.94, 95% CI 1.60–2.35). Heterogeneity was low (*I*^2^ = 6.9%; tau^2^ = 0.0028), indicating a consistent direction and magnitude of association across included estimates.

### Acute disease severity and respiratory support

Three studies contributed estimates for acute disease severity or respiratory support. Greater acute severity, including invasive mechanical ventilation or high-intensity respiratory support, was associated with higher odds of post-COVID syndrome (pooled OR 2.55, 95% CI 1.77–3.68). Heterogeneity was low to moderate (I^2^ = 13.0%; tau^2^ = 0.0139).

### ICU admission

Two studies contributed estimates for ICU admission. ICU admission was associated with higher odds of persistent post-COVID symptoms (pooled OR 1.88, 95% CI 1.24–2.83). No statistical heterogeneity was observed in this two-study synthesis (I^2^ = 0.0%; tau^2^ = 0.0000), although the small number of studies limits interpretation.

### Vaccination status

Two studies contributed estimates for vaccination status after harmonization of effect direction. No or incomplete vaccination was associated with higher odds of post-COVID syndrome compared with full vaccination (pooled OR 1.90, 95% CI 1.33–2.72). Heterogeneity was not observed (I^2^ = 0.0%; tau^2^ = 0.0000), but the evidence base was limited to two studies.

### Hypertension

Two studies contributed estimates for hypertension. Hypertension was associated with higher odds of post-COVID syndrome (pooled OR 1.60, 95% CI 1.16–2.20). Heterogeneity was low to moderate (I^2^ = 25.4%; tau^2^ = 0.0153). This association should be interpreted cautiously because of the small number of studies and possible residual confounding by age, comorbidity burden, and acute disease severity.

### Narrative synthesis of risk factors not eligible for pooling

Several candidate risk factors were extracted but not eligible for quantitative synthesis because estimates were incomplete, reported using non-comparable effect measures, or related to outcome definitions that were not directly comparable with the pooled analyses. These factors included obesity, age, smoking, dysgeusia, initial acute symptom load, neurologic or psychiatric comorbidity, and selected organ-specific abnormalities. These variables were therefore summarized narratively rather than combined statistically. These variables were therefore summarized narratively rather than combined statistically (Table 4).

**Table 4:** Candidate risk factors retained for narrative synthesis only.

Candidate factor	Reason for narrative synthesis/interpretation
Obesity/BMI >=30	PHOSP-COVID reported lower probability of full recovery among patients with obesity; retained qualitatively because complete CI was not available in the extraction table.
Age	Age was evaluated in several cohorts, but direction and comparability were inconsistent across outcome definitions and adjustment models.
Smoking	Active smoking was reported as a possible risk factor in one cohort but was not eligible for pooled analysis.
Dysgeusia	Reported by de Oliveira et al. as associated with Long COVID; retained as a symptom-specific qualitative predictor.
Initial symptom load	Ko et al. reported higher odds with increasing number of initial acute symptoms; retained qualitatively.
Neurologic/ psychiatric comorbidity	Reported in selected cohorts, including pediatric and neuropsychological outcome contexts; not pooled because definitions and estimates were heterogeneous.

### Sensitivity analyses

Leave-one-out sensitivity analyses showed that the direction of association remained unchanged after sequential exclusion of individual study-effect estimates. For female sex, the pooled OR after exclusion of one study ranged from 1.85 to 2.10. For acute disease severity or respiratory support, the pooled OR ranged from 2.19 to 2.98. For ICU admission, vaccination status, and hypertension, exclusion of one study left a single-study estimate; therefore, these analyses were interpreted only as descriptive stability checks rather than formal influence diagnostics. Detailed results of the leave-one-out sensitivity analyses are presented in Table 5.

**Table 5:** Leave-one-out sensitivity analysis by pooled risk-factor group.

Risk factor	Excluded study/effect	k retained	Pooled OR	95% CI	*I*^2^, %
Female sex	PHOSP-COVID (13)	3	2.1	1.71–2.58	0.0
Female sex	Pazukhina et al. (15)	3	1.88	1.38–2.55	32.6
Female sex	Nascimento et al. (16)	3	1.85	1.52–2.26	0.0
Female sex	Ko et al. (33)	3	1.93	1.50–2.50	37.9
Disease severity/respiratory support	PHOSP-COVID (13)	2	2.75	1.44–5.24	55.3
Disease severity/respiratory support	Rigoni et al. (18)	2	2.19	1.48–3.24	0.0
Disease severity/respiratory support	Lersritwimanmaen et al. (15)	2	2.98	1.80–4.94	18.5
ICU admission	Nascimento et al. (16)	1	2.6	1.11–6.10	0.0
ICU admission	de Oliveira et al. (20)	1	1.7	1.06–2.72	0.0
No/incomplete vaccination	Nascimento et al. (16)	1	2.08	1.10–3.95	0.0
No/incomplete vaccination	Lersritwimanmaen et al. (19)	1	1.82	1.18–2.81	0.0
Hypertension	Pazukhina et al. (15)	1	2.01	1.22–3.31	0.0
Hypertension	Ko et al. (33)	1	1.42	1.04–1.94	0.0

### Small-study effects and subgroup analyses

Formal assessment of small-study effects was not performed because fewer than 10 studies were available for each pooled risk-factor group. Funnel plots, if generated, should therefore be interpreted descriptively only. Formal subgroup meta-analysis and meta-regression were also not performed as primary analyses because most risk-factor groups included only two to four studies. Differences in operational outcome definition, follow-up duration, and geographic setting were considered qualitatively when interpreting the results.

Overall, the most reproducible risk factors for post-COVID syndrome among previously hospitalized patients were female sex and greater acute disease severity or respiratory support. ICU admission, no or incomplete vaccination, and hypertension were also associated with higher odds of post-COVID outcomes, although these findings were based on fewer studies and should be interpreted with greater caution.

## Discussion

### Principal findings

This systematic review and meta-analysis synthesized evidence on risk factors associated with post-COVID syndrome among patients previously hospitalized for COVID-19. Across the included hospital-based studies, the most consistently supported risk factors were female sex, greater acute disease severity or need for advanced respiratory support, ICU admission, incomplete or absent vaccination, and hypertension. Quantitative synthesis was possible for five risk-factor groups. Female sex was associated with higher odds of post-COVID outcomes, with a pooled OR of 1.94. Acute disease severity or respiratory support showed the strongest pooled association, with an OR of 2.55. ICU admission, incomplete or absent vaccination, and hypertension were also associated with increased odds of post-COVID syndrome, although these estimates were based on fewer studies.

Female sex was the most frequently pooled demographic predictor. Markers of acute severity, including invasive mechanical ventilation, high-flow oxygen, non-invasive ventilation, severe-critical acute illness, and ICU admission, formed the main clinical-severity domain. Other candidate factors, including obesity, age, smoking, dysgeusia, initial symptom load, and selected comorbidities, were reported in individual studies but could not be pooled because the available estimates were incomplete, differently defined, or insufficiently comparable.

### Interpretation of the main risk factors

The association between female sex and post-COVID outcomes was one of the most consistent findings in this review. This result is compatible with both hospital-based cohort evidence and broader Long COVID literature suggesting that women may be more likely to experience persistent symptoms, incomplete recovery, or multisystem symptom clusters after SARS-CoV-2 infection. Several explanations are possible, including sex-related differences in immune response, symptom perception and reporting, healthcare-seeking behavior, hormonal factors, and different distributions of autoimmune or dysautonomia-like phenotypes. However, the interpretation should remain cautious because female sex may also interact with age, baseline comorbidity, occupational exposure, and outcome ascertainment.

Acute disease severity and respiratory support showed the strongest association with subsequent post-COVID syndrome. This is clinically plausible. Patients with severe acute COVID-19 may experience more intense systemic inflammation, hypoxemia, endothelial injury, thromboinflammatory complications, organ damage, prolonged immobilization, and functional decline. In addition, the need for advanced respiratory support or invasive mechanical ventilation may identify patients at higher risk of post-intensive-care sequelae, respiratory impairment, physical deconditioning, and neuropsychological morbidity. Therefore, in hospitalized cohorts, post-COVID syndrome may partly reflect a combination of SARS-CoV-2-specific post-viral mechanisms and general consequences of severe acute illness.

ICU admission was also associated with higher odds of post-COVID symptoms. This association is biologically and clinically credible, but it is difficult to separate from acute disease severity, mechanical ventilation, length of hospital stay, and baseline comorbidity. ICU admission may act both as an independent marker of critical illness exposure and as a proxy for the most severe acute COVID-19 phenotype. Therefore, ICU-related findings should be interpreted as indicating a high-risk clinical subgroup rather than as evidence that ICU exposure alone causally produces post-COVID syndrome.

Vaccination status was another relevant factor. In the pooled analysis, no or incomplete vaccination was associated with higher odds of post-COVID outcomes compared with full vaccination. This finding is consistent with broader evidence suggesting that vaccination may reduce the risk of persistent post-COVID symptoms, either by lowering the probability of severe acute disease or by modifying viral replication, inflammatory burden, and immune activation during infection. However, the number of hospitalized studies contributing to this analysis was small. Moreover, vaccination status is closely related to calendar period, circulating variant, prior immunity, age, comorbidity profile, and changing admission criteria. Therefore, this association should be interpreted as suggestive rather than definitive in the hospitalized population. In addition, vaccination status is strongly linked to pandemic wave. Patients classified as unvaccinated or incompletely vaccinated were more likely to have been infected during earlier periods, when variants, admission thresholds, acute treatment protocols, and post-discharge follow-up systems differed from later phases of the pandemic. Therefore, the observed association may reflect both biological protection from vaccination and residual confounding by calendar period and healthcare context.

Hypertension was associated with increased odds of post-COVID syndrome, but the evidence was less robust than for female sex or acute severity. Hypertension may reflect underlying vascular, inflammatory, metabolic, or age-related vulnerability. At the same time, hypertension is strongly correlated with age, obesity, diabetes, cardiovascular disease, and severity of acute COVID-19. Therefore, residual confounding is likely. In this review, hypertension should be interpreted as a possible risk marker rather than a clearly independent causal predictor.

### Comparison with previous studies

The findings of this review are broadly consistent with larger syntheses of Long COVID risk factors showing that female sex, comorbidity burden, acute disease severity, previous hospitalization, and ICU admission are associated with increased risk of Long COVID or PCC (31). For example, broad population-level meta-analyses have identified hospitalization and ICU admission as important markers of increased post-COVID risk. However, direct comparison is challenging because many earlier syntheses combined hospitalized and non-hospitalized populations, included broader community-based samples, or focused primarily on symptom prevalence rather than risk factors in post-discharge cohorts. The present review therefore adds a narrower hospital-based synthesis by examining risk factors within previously hospitalized patients rather than comparing hospitalized and non-hospitalized patients as exposure groups.

The focus on previously hospitalized patients is an important distinction. In general infected populations, risk factors may reflect susceptibility to persistent symptoms after SARS-CoV-2 infection across a wide severity spectrum. In contrast, among hospitalized patients, predictors such as respiratory support, ICU admission, and severe-critical acute disease are particularly relevant because they capture the intensity of acute illness and possible overlap with post-intensive-care syndrome, respiratory impairment, physical deconditioning, and delayed recovery after severe systemic disease.

The association between female sex and post-COVID outcomes observed in this review is consistent with several cohort studies and broader Long COVID literature. However, the mechanisms remain uncertain and may include biological, immunological, hormonal, psychosocial, and ascertainment-related factors. Similarly, the association between acute severity or respiratory support and post-COVID outcomes is clinically plausible and consistent with post-discharge cohort evidence, but it may reflect both SARS-CoV-2-specific mechanisms and non-specific consequences of severe acute illness.

The vaccination findings are also consistent with broader evidence suggesting that vaccination may reduce the risk of Long COVID. Nevertheless, the hospitalized-population evidence remains limited. Vaccination status is closely linked to calendar period, variant wave, prior immunity, comorbidity profile, age, admission criteria, and changes in treatment standards. Therefore, the observed association between no or incomplete vaccination and post-COVID outcomes should be interpreted as suggestive and clinically relevant, but not sufficient to establish a fully independent causal effect in hospitalized cohorts.

### Outcome-definition heterogeneity

A central methodological issue in this review was the heterogeneity of post-COVID outcome definitions. The included studies used different labels, including post-COVID-19 condition, Long COVID, post-COVID syndrome, persistent symptoms, new or persistent symptoms, and incomplete recovery. More importantly, they used different operational definitions. Some studies applied WHO post-COVID-19 condition, others used at least one persistent symptom at a fixed follow-up point, structured interview-defined Long COVID, or patient-perceived non-recovery.

Relapsing or fluctuating symptoms were included only when they formed part of the original study-defined post-COVID outcome, but they could not be analyzed as a distinct outcome because comparable risk-factor estimates for relapsing symptom trajectories were not reported.

Recent consensus and methodological literature has emphasized that PCC research remains affected by inconsistent definitions, lack of objective diagnostic tests, variable symptom attribution, and incomplete exclusion of alternative diagnoses (32). These issues are particularly important for risk-factor research because the magnitude and direction of associations may differ depending on whether the outcome is defined as WHO-PCC, any persistent symptom, patient-perceived incomplete recovery, or a domain-specific sequela. Consequently, pooled estimates in the present review should be interpreted as associations with study-defined post-COVID outcomes rather than with a single uniformly validated diagnostic entity.

This heterogeneity matters because risk-factor associations may differ depending on how the outcome is defined. A broad definition such as “at least one persistent symptom” may capture a wider and more heterogeneous group of patients than a definition requiring symptoms lasting at least 3 months, functional impairment, or exclusion of alternative diagnoses. Similarly, patient-perceived incomplete recovery may reflect global functional status, while symptom-specific outcomes may capture particular domains such as fatigue, dyspnea, anxiety, depression, or pulmonary impairment. For this reason, this review extracted both the author-reported label and the operational outcome definition and interpreted pooled estimates according to operational comparability rather than terminology alone.

### Clinical and public health implications

The findings support a risk-based approach to post-discharge follow-up after COVID-19 hospitalization. Patients with severe acute disease, ICU admission, advanced respiratory support, or other markers of increased post-COVID risk may warrant more structured post-discharge assessment. Vaccination status may also help contextualize risk, although it should be interpreted alongside age, comorbidity, variant period, and severity of acute illness. Female patients and those with hypertension or cardiometabolic comorbidity may also require careful monitoring, especially if they report persistent fatigue, dyspnea, cognitive symptoms, psychological symptoms, or reduced functional capacity.

In clinical practice, these risk factors should not be used as a simple deterministic prediction rule. Rather, they can help identify groups in whom systematic symptom screening, functional assessment, pulmonary evaluation, cardiovascular assessment, mental health screening, and rehabilitation referral may be particularly important. The results also suggest that post-COVID pathways should be integrated with broader post-hospitalization and post-ICU care, because part of the long-term morbidity after severe COVID-19 may overlap with post-intensive-care syndrome and recovery after severe respiratory illness.

From a health-system perspective, the findings may help prioritize post-COVID services for high-risk discharged patients. Hospital discharge protocols could include structured follow-up for patients with severe acute disease, ICU admission, mechanical ventilation, or persistent symptoms at discharge. However, implementation should remain flexible because local patient populations, vaccination coverage, variant periods, and healthcare resources vary substantially.

### Strengths and limitations

This review has several strengths. It focused specifically on patients hospitalized for COVID-19, a clinically important population at increased risk of persistent morbidity and post-discharge functional impairment. The review also separated author-reported terminology from operational outcome definitions, which is essential in post-COVID research because the same terms are often applied to different diagnostic thresholds, symptom durations, and ascertainment methods. In addition, both qualitative and quantitative risk-factor evidence were extracted, effect directions were harmonized so that pooled estimates consistently represented higher odds of adverse post-COVID outcomes, and meta-analyses were performed only when effect estimates were sufficiently comparable. Methodological quality was assessed using a Newcastle-Ottawa Scale-based approach, and leave-one-out sensitivity analyses were conducted for pooled risk-factor groups.

Several limitations should also be acknowledged. The most important limitation is the small number of studies available for each pooled risk-factor group. Although the review included several hospital-based cohorts, most pooled analyses were based on only two to four studies. Therefore, the pooled estimates should be interpreted as evidence summaries and risk signals rather than definitive prediction parameters. The small number of studies also prevented reliable formal assessment of publication bias, meta-regression, and subgroup analyses.

A second major limitation is heterogeneity in post-COVID outcome definitions. Included studies differed in terminology, follow-up duration, symptom ascertainment, functional impairment criteria, attribution rules, and the extent to which alternative diagnoses were assessed or excluded. Symptoms such as fatigue, dyspnea, cognitive impairment, sleep disturbance, anxiety, and reduced exercise tolerance overlap with depression, anxiety, deconditioning, cardiopulmonary disease, post-intensive-care syndrome, and other conditions that may emerge or worsen after hospitalization or during the pandemic independently of SARS-CoV-2 infection. This may have introduced outcome misclassification and limited comparability across studies.

A third limitation concerns exposure classification and study populations. The inclusion of probable COVID-19 cases may have introduced exposure misclassification, although in early high-transmission hospital settings the pre-test probability of true SARS-CoV-2 infection was high. In addition, hospitalization was not a uniform exposure across countries or pandemic waves. Admission thresholds may have varied according to health-system capacity, local clinical practice, isolation policies, availability of outpatient care, and social circumstances. During periods of health-system pressure, some patients who would ordinarily have been hospitalized may have remained in the community, whereas in other settings hospitalization may have been influenced by monitoring needs, isolation requirements, or lack of home support. Therefore, hospitalization should be interpreted as a pragmatic clinical marker rather than a perfectly standardized indicator of acute disease severity.

A fourth limitation is residual confounding. Adjustment strategies differed substantially across studies, and although adjusted estimates were prioritized, residual confounding remains likely, particularly for factors such as hypertension, obesity, ICU admission, acute disease severity, respiratory support, and vaccination status. Calendar period is also important because admission criteria, circulating SARS-CoV-2 variants, vaccination coverage, treatment standards, and post-discharge follow-up systems changed over time. These factors may have influenced both the probability of hospitalization and the risk or detection of post-COVID outcomes.

A fifth limitation is that not all candidate risk factors could be included in quantitative synthesis. Several factors, including obesity, age, smoking, dysgeusia, initial symptom load, neurologic or psychiatric comorbidity, organ-specific abnormalities, length of hospital stay, and treatment-related variables were retained for narrative synthesis because effect estimates were incomplete, reported using different measures, or linked to non-comparable outcome definitions. Treatment-related and hospital-management variables, including pharmacological agents, corticosteroids, anticoagulation, rehabilitation, oxygen therapy, ICU-specific interventions, respiratory-support modalities, and length of hospital stay, were inconsistently defined and strongly confounded by indication and acute disease severity. Therefore, the review should not be interpreted as evaluating the effectiveness or harms of specific acute-phase interventions. The findings are more directly applicable to post-discharge risk stratification than to treatment-selection decisions during acute COVID-19 hospitalization.

Finally, the review was limited to studies published in English or Russian, which may have introduced language bias and reduced the representation of evidence from some regions. The review protocol was not prospectively registered, which may affect reproducibility and should be considered a methodological limitation. However, the eligibility criteria, data extraction framework, risk-factor grouping, and statistical approach were defined before the quantitative synthesis was conducted.

### Future research

Future studies should use standardized post-COVID outcome definitions and report operational criteria in detail. At minimum, studies should specify the time from acute infection or discharge, symptom duration, symptom assessment instrument, functional impairment requirement, attribution to SARS-CoV-2 infection, exclusion of alternative diagnoses, and whether baseline symptom status was available.

Future risk-factor studies should report adjusted effect estimates with 95% confidence intervals for a common set of predictors, including sex, age, comorbidities, vaccination status, acute severity, oxygen requirement, ICU admission, mechanical ventilation, length of stay, and symptom burden at discharge. Adjustment models should be clearly described, and studies should distinguish between predictors of any persistent symptom and predictors of functionally significant post-COVID syndrome. Larger prospective cohorts with harmonized follow-up would allow more reliable subgroup analyses and development of validated risk-prediction models for post-discharge care.

## Conclusion

In this systematic review and meta-analysis of patients previously hospitalized for COVID-19, the most consistently supported risk factors for post-COVID syndrome were female sex, greater acute disease severity or need for advanced respiratory support, ICU admission, incomplete or absent vaccination, and hypertension. The clearest pooled association was observed for acute disease severity or respiratory support, while female sex, ICU admission, no or incomplete vaccination, and hypertension also showed positive associations. However, because several pooled analyses were based on only two studies, these estimates should be interpreted as risk signals rather than definitive prediction parameters.

The findings suggest that post-COVID syndrome after hospitalization is not explained by a single mechanism. Instead, it likely reflects the interaction of patient-level vulnerability, acute disease severity, intensive-care exposure, respiratory support, comorbidity burden, and post-discharge recovery trajectories. For clinical practice, these results support risk-based follow-up of patients discharged after COVID-19 hospitalization, particularly those with severe acute disease, ICU admission, advanced respiratory support, or incomplete vaccination.

However, the evidence remains limited by heterogeneous outcome definitions, small numbers of studies per risk factor, differing adjustment strategies, and residual confounding. Future studies should use standardized operational definitions of post-COVID syndrome, harmonized follow-up protocols, and consistent reporting of adjusted effect estimates. Such data are needed to develop reliable risk stratification tools and to guide targeted post-COVID care for previously hospitalized patients.

## Data Availability

The original contributions presented in the study are included in the article/Supplementary material, further inquiries can be directed to the corresponding authors.
