# Supplementary material for: Risk factors for post-COVID-19 condition among previously hospitalized COVID-19 patients: a systematic review and meta-analysis
Source: Front Public Health. 2026 Aug 14;14:1911482. doi: 10.3389/fpubh.2026.1911482 (PMC13521914; doi:10.3389/fpubh.2026.1911482)
Supplement: Supplementary file 1 [file Table_1.docx]

**Supplementary Table S1. Full database-specific search strategies**

| Database | Search string |
| --- | --- |
| PubMed/MEDLINE | (("COVID-19"[Mesh] OR "SARS-CoV-2"[Mesh] OR COVID-19[tiab] OR "coronavirus disease 2019"[tiab] OR SARS-CoV-2[tiab]) AND ("post-COVID-19 condition"[tiab] OR "post COVID-19 condition"[tiab] OR "long COVID"[tiab] OR "post-COVID syndrome"[tiab] OR PASC[tiab] OR "persistent symptoms"[tiab] OR "post-acute sequelae"[tiab]) AND (hospitalized[tiab] OR hospitalised[tiab] OR hospitalization[tiab] OR hospitalisation[tiab] OR discharged[tiab] OR ICU[tiab] OR "intensive care"[tiab]) AND ("risk factor"[tiab] OR predictor*[tiab] OR determinant*[tiab] OR association*[tiab] OR odds ratio[tiab] OR multivaria*[tiab])) AND ("2020/01/01"[Date - Publication] : "2026/02/24"[Date - Publication]) |
| Scopus | TITLE-ABS-KEY(("COVID-19" OR "coronavirus disease 2019" OR "SARS-CoV-2") AND ("post-COVID-19 condition" OR "post COVID-19 condition" OR "long COVID" OR "post-COVID syndrome" OR PASC OR "persistent symptoms" OR "post-acute sequelae") AND (hospitalized OR hospitalised OR hospitalization OR hospitalisation OR discharged OR ICU OR "intensive care") AND ("risk factor" OR predictor* OR determinant* OR association* OR "odds ratio" OR multivaria*)) AND PUBYEAR > 2019 AND PUBYEAR < 2027 |
| Web of Science Core Collection | TS=(("COVID-19" OR "coronavirus disease 2019" OR "SARS-CoV-2") AND ("post-COVID-19 condition" OR "post COVID-19 condition" OR "long COVID" OR "post-COVID syndrome" OR PASC OR "persistent symptoms" OR "post-acute sequelae") AND (hospitalized OR hospitalised OR hospitalization OR hospitalisation OR discharged OR ICU OR "intensive care") AND ("risk factor" OR predictor* OR determinant* OR association* OR "odds ratio" OR multivaria*)); Timespan=2020-2026 |
| WHO COVID-19 Global Literature Database | ("post-COVID-19 condition" OR "long COVID" OR PASC OR "persistent symptoms" OR "post-acute sequelae") AND (hospitalized OR hospitalised OR discharged OR ICU OR "intensive care") AND ("risk factor" OR predictor* OR determinant* OR association* OR "odds ratio" OR multivaria*) |

**Supplementary Table S2. Full characteristics of included studies**

| Study ID | Study | Country | Design | Population | Analytic sample | Outcome definition | Follow-up | Main candidate risk factors | Use in this review | NOS score | Risk category |
| --- | --- | --- | --- | --- | --- | --- | --- | --- | --- | --- | --- |
| S01 | PHOSP-COVID Collaborative Group 2022 | United Kingdom | Prospective multicentre cohort | Adults discharged after COVID-19 hospitalization | 2320 at 5-month visit; 804 with 1-year recovery assessment | Patient-perceived recovery (not fully recovered vs recovered); symptom burden and clusters | 5 months and 1 year | Female sex; BMI ≥30; IMV/WHO severity 7-9; systemic corticosteroids | Female sex and IMV used in pooled analyses; obesity retained qualitatively because CI not available in working extraction | 7 | Low-moderate |
| S02 | Pazukhina et al. 2022 (StopCOVID) | Russia (Moscow) | Prospective cohort | Adults and children after hospital discharge | Adults n=1013; children n=360 at both follow-up time points | WHO post-COVID-19 condition (PCC) | 6 and 12 months after discharge | Female sex; hypertension; pediatric neurologic and allergic respiratory comorbidities | Adult female sex (6 months) and hypertension (12 months) used in pooled analyses | 6 | Moderate |
| S03 | Nascimento et al. 2023 | Brazil | Observational cohort | Adults discharged from 2 hospitals | 412 | ≥1 persistent symptom | 90 days after discharge | Female sex; ICU admission; no/incomplete vaccination | All three retained in pooled analyses | 6 | Moderate |
| S04 | Rigoni et al. 2022 | Italy | Prospective cohort | Hospitalized adults after discharge | 413 analyzed after discharge (from 471 hospitalized) | Any persistent symptom | 6 and 12 months | Need for HFNC/NIV/IMV; acute severity markers | Respiratory support category (6-month model) retained for pooled severity analysis | 6 | Moderate |
| S05 | Lersritwimanmaen et al. 2025 | Thailand | Ambidirectional cohort | Previously hospitalized adults | 295 | Long COVID by structured interview | 3 months, 1 year and 2 years | Severe-critical acute illness; vaccination status | Severe-critical illness and vaccination retained in pooled analyses | 6 | Moderate |
| S06 | Bai et al. 2021 | Italy (Milan) | Prospective cohort | Hospitalized adults from 2 hospitals | 377 | Long COVID = ≥1 physical or psychological symptom | >4 weeks after recovery | Female sex; age; active smoking; severity proxies | Used qualitatively; CIs unavailable in the working extraction table | 6 | Moderate |
| S07 | de Oliveira et al. 2022 | Brazil | Cross-sectional follow-up | Hospitalized adults | 439 | Long COVID = ≥1 symptom lasting ≥4 weeks | 2-12 months after onset | ICU admission; dysgeusia | ICU admission retained in pooled analyses; dysgeusia kept qualitatively | 5 | Moderate-high |
| S08 | Ko et al. 2022 | France | Prospective cohort | Hospitalized adults including ICU transfers | 316 | Persistent symptoms / long COVID | 4 months after hospitalization | Female sex; hypertension; number of initial symptoms | Female sex and hypertension retained in pooled analyses; symptom load kept qualitatively | 6 | Moderate |
| S09 | Dryden et al. 2022 | South Africa | Prospective national cohort | Adults after COVID-19 hospitalization | 1873 at 3-month follow-up | New or persistent symptoms | 3 months after discharge | Female sex; ICU admission; oxygen therapy proxies | Used qualitatively because reported measure was aIRR and not pooled with OR-based synthesis | 7 | Low-moderate |
| S10 | Mandal et al. 2021 | United Kingdom | Prospective clinic follow-up | Adults discharged after hospitalization | 384 | Persistent symptoms with biomarker / imaging follow-up | Median 54 days after discharge | Persistent fatigue, dyspnea, D-dimer, CRP, abnormal chest X-ray | Descriptive/clinical context only; no pooled adjusted effect estimate extracted | 5 | Moderate-high |
| S11 | Huang et al. 2021/2023 | China (Wuhan) | Ambidirectional cohort | Adults discharged after hospitalization | 1733 | ≥1 symptom and domain-specific sequelae | ~6 months after symptom onset | Severe acute disease; female sex for several domains; organ-specific outcomes | Used qualitatively because outcome was not a single overall PCC endpoint | 7 | Low-moderate |

**Supplementary Table S3. Extracted effect estimates and inclusion in pooled analyses**

| Effect ID | Study ID | Study | Country | Outcome definition | Follow-up | Risk factor | Comparison | Effect measure | Estimate | Lower 95% CI | Upper 95% CI | Included in pooled analysis | Pooled factor group | Notes |
| --- | --- | --- | --- | --- | --- | --- | --- | --- | --- | --- | --- | --- | --- | --- |
| EF01 | S01 | PHOSP-COVID 2022 | United Kingdom | Not fully recovered (inverse of recovery OR) | 1 year | Female sex | Female vs male | OR | 1.47 | 1.01 | 2.17 | Yes | Female sex | Converted from reported OR for recovery (1/0.68) |
| EF02 | S01 | PHOSP-COVID 2022 | United Kingdom | Not fully recovered (inverse of recovery OR) | 1 year | Mechanical ventilation / very severe acute disease | IMV or WHO severity 7-9 vs lower severity | OR | 2.38 | 1.32 | 4.35 | Yes | Disease severity / respiratory support | Converted from reported OR for recovery (1/0.42) |
| EF03 | S01 | PHOSP-COVID 2022 | United Kingdom | Not fully recovered (inverse of recovery OR) | 1 year | Obesity | BMI ≥30 vs <30 | OR | 2.0 |  |  | No | Qualitative only | CI not available in working extraction |
| EF04 | S02 | Pazukhina 2022 (StopCOVID) | Russia | WHO PCC | 6 months | Female sex | Female vs male | OR | 2.04 | 1.57 | 2.65 | Yes | Female sex | Adult cohort |
| EF05 | S02 | Pazukhina 2022 (StopCOVID) | Russia | WHO PCC | 12 months | Female sex | Female vs male | OR | 2.04 |  |  | No | Qualitative only | Repeated within-study time point not used in main pooled model |
| EF06 | S02 | Pazukhina 2022 (StopCOVID) | Russia | WHO PCC | 12 months | Hypertension | Hypertension vs no hypertension | OR | 1.42 | 1.04 | 1.94 | Yes | Hypertension | Adult cohort |
| EF07 | S03 | Nascimento 2023 | Brazil | ≥1 persistent symptom | 90 days | Female sex | Female vs male | OR | 2.44 | 1.57 | 3.76 | Yes | Female sex | Adjusted estimate |
| EF08 | S03 | Nascimento 2023 | Brazil | ≥1 persistent symptom | 90 days | ICU admission | ICU vs no ICU | OR | 1.7 | 1.06 | 2.71 | Yes | ICU admission | Adjusted estimate |
| EF09 | S03 | Nascimento 2023 | Brazil | ≥1 persistent symptom | 90 days | Vaccination status | No/incomplete vaccination vs full vaccination | OR | 1.82 | 1.18 | 2.81 | Yes | Vaccination status | Adjusted estimate |
| EF10 | S04 | Rigoni 2022 | Italy | Any persistent symptom | 6 months | Disease severity / respiratory support | HFNC/NIV/IMV vs low-flow/no oxygen | OR | 4.0 | 1.99 | 8.05 | Yes | Disease severity / respiratory support | Adjusted estimate |
| EF11 | S04 | Rigoni 2022 | Italy | Any persistent symptom | 12 months | Disease severity / respiratory support | HFNC/NIV/IMV vs low-flow/no oxygen | OR | 3.66 |  |  | No | Qualitative only | CI not available in working extraction |
| EF12 | S05 | Lersritwimanmaen 2025 | Thailand | Long COVID | 3 months | Disease severity | Severe-critical vs mild-moderate acute illness | OR | 2.06 | 1.23 | 3.46 | Yes | Disease severity / respiratory support | Adjusted estimate |
| EF13 | S05 | Lersritwimanmaen 2025 | Thailand | Long COVID | 3 months | Vaccination status | Unvaccinated vs vaccinated | OR | 2.08 | 1.11 | 4.0 | Yes | Vaccination status | Derived as inverse of protective vaccination effect |
| EF14 | S06 | Bai 2021 | Italy | Long COVID = ≥1 symptom | >4 weeks | Female sex | Female vs male | OR | 3.3 |  |  | No | Qualitative only | CI not available in working extraction |
| EF15 | S06 | Bai 2021 | Italy | Long COVID = ≥1 symptom | >4 weeks | Age | Per 10-year increase | OR | 1.03 |  |  | No | Qualitative only | CI not available in working extraction |
| EF16 | S06 | Bai 2021 | Italy | Long COVID = ≥1 symptom | >4 weeks | Active smoking | Smoker vs non-smoker | OR |  |  |  | No | Qualitative only | Direction reported as increased risk |
| EF17 | S07 | de Oliveira 2022 | Brazil | Long COVID = ≥1 symptom lasting ≥4 weeks | 2-12 months | ICU admission | ICU vs no ICU | OR | 2.6 | 1.19 | 6.56 | Yes | ICU admission | Adjusted estimate |
| EF18 | S07 | de Oliveira 2022 | Brazil | Long COVID = ≥1 symptom lasting ≥4 weeks | 2-12 months | Dysgeusia | Present vs absent | OR | 2.0 |  |  | No | Qualitative only | CI not available in working extraction |
| EF19 | S08 | Ko 2022 | France | Persistent symptoms / long COVID | 4 months | Female sex | Female vs male | OR | 1.94 | 1.17 | 3.22 | Yes | Female sex | Adjusted estimate |
| EF20 | S08 | Ko 2022 | France | Persistent symptoms / long COVID | 4 months | Hypertension | Hypertension vs no hypertension | OR | 2.01 | 1.22 | 3.31 | Yes | Hypertension | Adjusted estimate |
| EF21 | S08 | Ko 2022 | France | Persistent symptoms / long COVID | 4 months | Initial symptom load | Per additional acute symptom | OR | 1.35 |  |  | No | Qualitative only | CI not available in working extraction |
| EF22 | S09 | Dryden 2022 | South Africa | New or persistent symptoms | 3 months | Female sex | Female vs male | aIRR | 1.2 |  |  | No | Qualitative only | Different effect metric; not pooled |
| EF23 | S09 | Dryden 2022 | South Africa | New or persistent symptoms | 3 months | ICU admission | ICU vs no ICU | aIRR | 1.17 |  |  | No | Qualitative only | Different effect metric; not pooled |
| EF24 | S11 | Huang 2021/2023 | China | Fatigue/muscle weakness | 6 months | Severe acute disease | Scale 5-6 vs scale 3 | OR | 2.75 |  |  | No | Qualitative only | Outcome domain-specific, not overall PCC |

**Supplementary Table S4. Newcastle-Ottawa Scale-based risk-of-bias assessment**

| Study ID | Study | Selection | Comparability/representativeness | Outcome assessment | Follow-up/response completeness | Total score | Risk category | Main concerns |
| --- | --- | --- | --- | --- | --- | --- | --- | --- |
| S01 | PHOSP-COVID 2022 | 3 | 1 | 2 | 1 | 7 | Low-moderate | Some attrition at 1-year follow-up; outcome partly self-reported |
| S02 | Pazukhina 2022 (StopCOVID) | 2 | 1 | 2 | 1 | 6 | Moderate | Telephone follow-up; participation rate below 50% of discharged patients |
| S03 | Nascimento 2023 | 2 | 1 | 2 | 1 | 6 | Moderate | Single-country cohort with moderate sample size |
| S04 | Rigoni 2022 | 2 | 1 | 2 | 1 | 6 | Moderate | Post-discharge loss from original hospitalized cohort |
| S05 | Lersritwimanmaen 2025 | 2 | 1 | 2 | 1 | 6 | Moderate | Single setting; interview-based outcome ascertainment |
| S06 | Bai 2021 | 2 | 1 | 2 | 1 | 6 | Moderate | Early-pandemic cohort and broad outcome definition |
| S07 | de Oliveira 2022 | 2 | 1 | 1 | 1 | 5 | Moderate-high | Cross-sectional follow-up; broad symptom definition |
| S08 | Ko 2022 | 2 | 1 | 2 | 1 | 6 | Moderate | Moderate size; persistent-symptom outcome based on follow-up assessment |
| S09 | Dryden 2022 | 3 | 1 | 2 | 1 | 7 | Low-moderate | National cohort but self-reported symptoms |
| S10 | Mandal 2021 | 2 | 1 | 1 | 1 | 5 | Moderate-high | Clinic follow-up cohort with potential referral bias |
| S11 | Huang 2021/2023 | 3 | 1 | 2 | 1 | 7 | Low-moderate | Strong cohort design; domain-specific rather than single PCC outcome |
